# LLM tool use in manuscript review: Toward guidance and transparency

**DOI:** 10.26508/lsa.202603913

**Published:** 2026-11-10

**Authors:** Chi-Ping Day, Besa Xhabija, Vishaka Gopalan

**Affiliations:** 1 Cancer Data Science Laboratory, Center for Cancer Research, National Cancer Institute, National Institutes of Health , Bethesda, MD, USA; 2 Department of Natural Science, College of Arts, Sciences and Letters, University of Michigan–Dearborn , Dearborn, MI, USA

## Abstract

The peer review system is straining under rising submissions, growing manuscript complexity, and a shrinking reviewer pool. Meanwhile an increasing number of researchers now use tools based on large language models (LLMs) when performing peer review, often without disclosure and against journal policies. This erodes trust in peer review and risks deteriorating the quality and integrity of research publications. We argue for professionalization and transparency rather than prohibition, and propose guidelines built on three aspects: model selection and data protection, human guidance and accountability, and publication integrity. These principles offer a sustainable path forward, leaving scientific judgment where it belongs; that is, with human experts.

## Abstract (precis)

Reviewers increasingly use LLM-based tools to evaluate manuscripts without disclosure, risking degraded rigor and privacy violations. This commentary argues LLM use in peer review needs professionalism and ethical guardrails, and proposed paths toward transparent, responsible adoption.

## Introduction

Peer review is undergoing a transformation. Across laboratories, editorial offices, and conference program committees, reviewers are turning to tools based on large language models (LLM agents) to help them read, evaluate, and comment on manuscripts, often without disclosure. However, using such tools but ignoring its technical issues and risks can result in the degradation of scientific values or even legal fights in data privacy. The history of human innovation has demonstrated that the dissemination of a tool requires earning the community’s trust. Only when the use of technology is understood and disclosed will people trust it enough to deploy and improve it responsibly. This principle applies to use of LLM agents in peer review process, too. If left unchecked, this unmonitored status quo has the potential to erode the basic confidence of scientific publication, human critical thinking, and the long-term retention of scientific knowledge. In Issac Asimov’s *Robot* series from the 1950s, each robot model is a specific, documented unit built for a defined function, vetted before deployment, and follows the same ethic rules without sparing human responsibility. This should be applied to the tools for peer review process today. Now is a critical time for authors, editors, reviewers, and funding agencies to examine the challenges in the current publishing system, the incentives and issues of using LLM agents, and the plausible solutions.

## What Are the Problems and Why Have They Emerged Now

The peer-review system is buckling under a set of pressures that have been building for years. One analysis found that the total number of indexed articles rose by roughly 47% between 2016 and 2022 alone, a pace of growth that has outstripped any corresponding increase in the number of practicing scientists available to review that work (Hanson et al, 2024). Editors describe a parallel strain on the reviewer pool itself. For instance, one journal editor-in-chief recently noted that his office now has to send out roughly twice as many reviewer invitations as a decade ago to secure a single completed review (Holmstrup, 2024). The result is a widening gap between the supply of willing, qualified reviewers and the volume of manuscripts that need evaluation.

At the same time, manuscripts themselves have grown more complex. Studies are increasingly multidisciplinary. A single paper might combine cell biology, computational modeling, genomic analysis, and clinical data, stretching well beyond the expertise of any one reviewer. Reading multidisciplinary manuscripts overwhelms reviewers from a single area of expertise. Journals may try to assign several specialists, but this requires knowledge of different research fields and coordination, so that individual reviewers can focus on the section relevant to their training. Each reviewer needs to spend more time studying the report, which delays the review process and complicates the integration of multiple review reports. It may eventually be unrealistic to expect that every manuscript will be examined and analyzed extensively at the review stage, and fabricated or inaccurate references and unverified statistical claims may slip through the cracks between reviewer and editorial checks.

Faced with this mismatch between demand and capacity, many reviewers have turned to general-purpose LLMs like ChatGPT, Claude, or Gemini to help draft or accelerate their reviews, usually without disclosing it. A global survey of more than 1,600 researchers found that over half reported using LLM-based tools for review tasks in 2025, up sharply from the prior year, despite many journals explicitly prohibiting the practice (Naddaf, 2026). At a major machine-learning conference, some attendees refused to let LLM agents review their own submissions, while using it anyway to review other people’s papers without disclosure (Brainard, 2026). This contradiction captures the underlying dynamic well: researchers recognize LLM’s usefulness but fear being professionally discredited if they admit to using it.

This silence carries real risks. Using LLM agents without appropriate training or domain knowledge can introduce bias or inaccuracy into reviews, leaving reviewers themselves, editors, and readers unaware. If multiple reviewers consult similar models, their reviews may converge for reasons unrelated to scientific importance, but due to shared model-level habits and blind spots (Hou et al, 2006). Uploading an unpublished manuscript to a general commercial LLM service also raises data-security concerns, though enterprise and academic-institutional subscriptions may offer contractual protection. Furthermore, lack of transparency risks creating a hypothetical scenario in which LLM-generated manuscripts are evaluated largely by LLM review tools, with neither side optimized for genuine scientific vetting. Perhaps most corrosively, undisclosed use produces a collective open secret, a situation in which nearly everyone involved understands that LLM agents are being used inappropriately, yet the system carries on regardless. Eventually, this erosion of trust threatens to undermine confidence in the peer review system as a whole.

## The Urgent Need of Professional LLM Agents for Quality Assurance in Review Processes

The right response to this situation is not to try to stamp out LLM agent use in peer review. Under the pressures described above, that would drive more use underground. Instead, stakeholders need to bring such tools into the light and set standards for it, with the goal of preserving the integrity and quality of the review process while capturing the genuine efficiency gains that LLM agent can offer.

Reporting in *Science* magazine underscores why some distinctions matter more than others: current evidence suggests LLM agents are comparatively strong at routinized, tedious verification work that overworked human reviewers often skip, such as checking and validating calculations, consistence of text and figures, cited sources, and submitted code (Brainard, 2026). By contrast, general-purpose models without appropriate context may misjudge conventions specific to subdiscipline, make unreasonable requests for additional experiments, produce excessively long and poorly prioritized reviews, and create biases inherited from their training data. One recent controlled audit, for example, found that several current general-purpose LLMs consistently rated otherwise-identical manuscripts more favorably when the authors were attributed to higher-ranked institutions (Vasu et al, 2026). Since general-purpose LLMs are trained on existing literature, they may also be more likely to favor work that fits established patterns in a field, which could systematically disadvantage paradigm-shifting research if left unchecked.

These are the limitations of present-day models (Bhardwaj, 2025), but model capabilities in this area are moving quickly. The practical implication is not that general-purpose LLM is permanently unsuited to review, but that any given model’s suitability for a given task needs to be demonstrated and periodically reassessed, rather than assumed. A model is likely to be more trustworthy for mechanical verification work than for judgments requiring field-specific norms or an assessment of a study’s novelty and significance, but where exactly that line falls will keep shifting, and guidelines should be built to be revisited rather than fixed. Afterall, an LLM agent is an expert system, not a scholar; even as that gap narrows, the responsibility for judging a manuscript’s scientific value, novelty, and significance should remain a human one.

## What Authors Want Matters Too

Any framework for LLM agent-assisted review should also account for how authors feel about it, since they bear the consequences of how LLM agent is used in evaluating their manuscripts. A relevant data point comes from a recent EMBO Reports survey of researchers who had experienced both LLM-generated and standard human peer review of their own manuscripts: researchers who compared LLM-generated reviews with ordinary human peer review overwhelmingly preferred using LLM agent as a self-checking tool they could apply to their own work before submission, rather than as a substitute for human reviewers evaluating them (Lemberger et al, 2026). This suggests that authors are not simply opposed to LLM agent involvement in review as a matter of principle; in contrast, they want a say in how and when it is applied to their own work, and they are more comfortable with LLM agents when they control its use themselves. That preference for author agency is a strong argument for the disclosure-and-consent principles discussed below: LLM agent use in peer review should not just be professionalized for reviewers' sake, but structured so it isn’t imposed on authors without their knowledge or input.

## Plausible Solutions

### Establishing clear, co-developed guidelines

The first step toward normalizing use of LLM agents in manuscript review is to build a set of well-defined guidelines, developed jointly by editors, reviewers, and authors, that address several aspects together as a single coherent standard. Three aspects of LLM agent-assisted peer review, in our view, deserve particular attention: (1) professionalism and data protection; (2) human primacy and accountability; and (3) integrity of the scientific publication system.

First, any LLM agent used in review should have documented capabilities and limitations that reviewers understand, along with appropriate data-privacy protections, whether that comes from a specialized review tool or from a general-purpose model used under an institutional or enterprise agreement with adequate confidentiality terms. Protection measures may include data encryption, insulation of the manuscript from model training, and certification of cybersecurity infrastructure.

Second, guidelines need to define reviewer responsibility. Reviewers who use LLM agent bear direct accountability for that use and must disclose it. Their use of LLM agents should be limited to quality-assurance functions — such as writing and typing support, consistency checks, assessment of statistical power, and evaluation of chosen methods — while reviewers personally review and justify every claim or suggestion a LLM agent generates before relying on it and attach the LLM-generated output to the final report as a matter of transparency. The reviewer remains fully responsible for the content, accuracy, fairness, and final recommendations of the review regardless of what tool assisted in preparing it.

Third, editors have an obligation to oversee LLM assistance in the process and to ensure that any disclosed use of LLM agent is communicated to authors as part of the review. Guidelines should give authors the right to know, and where appropriate to object, before their unpublished work is processed by an LLM agent — a point reinforced by the EMBO Reports finding above, that authors value having a say in how the LLM agent is applied to their own manuscripts. In practice, we envision this taking the form of a short standard notice that journals could send to reviewers at the point of invitation: informing them the features of tools that are permitted, specifying that such tools may only be used for the quality-assurance functions, requiring that any LLM-generated output be disclosed, and reiterating that reviewers remain fully and personally responsible for their review. The information in the notice can be provided to authors as part of disclosure. Such a notice would be one of the practical ways for the editorial office to apply these guidelines, and individual journals and publishers remain best placed to decide the form such disclosures should take for their own authors and reviewers.

### Strengthening infrastructure and support for the use of LLM agents

This shift in the peer-review system needs the support of scientific publishers. In addition to making clear policies on how LLM agent use is and is not permitted in review, in the long term, publishers and funding agencies should work together to identify LLM-based review tools that meet the criteria above, whatever their underlying model. Ideally, funding agencies and/or publishers can cover the cost of using a vetted tool when a reviewer chooses to do so, removing the financial burden that might otherwise push reviewers toward unregulated free tools. These are initial steps to establish structural guardrails around LLM agent use, to prevent science from defaulting into algorithmic repetition, and to preserve the human evaluation essential for scientific progress.

There are precedents for publishers experimenting in this direction already. A major conference on machine learning applied a coordinated team of language models to help process tens of thousands of submissions, pairing each with at least two human reviews and asking judges to weigh both (Biswas et al, 2026 *Preprint*). A medical journal affiliated with the *New England Journal of Medicine* has piloted a seven-day “fast track” combining LLM-generated analysis with human editorial review for papers likely to be accepted through the normal process (Manrai et al, 2026). A preprint server has begun offering authors the option to route their own manuscripts through confidential LLM-based technical-review checkers before submission (Zhao, 2025). None of these initiatives treat LLM agent as a replacement for human judgment; each treats it as a legitimate, disclosed part of the process rather than something to be hidden.

## Open Questions Requiring Further Discussion

Several issues remain unresolved and deserve continued discussion among researchers, publishers, and funders:

### Detection

How can undisclosed LLM agent use in a review be reliably identified after the fact, given how fluent LLM-generated text has become?

### Domain specialization

Which research field is a given LLM agent actually competent to evaluate, and who validates that competence before a tool is trusted, and how often should that validation be repeated as models change? Even for research in the same category (e.g., cancer research), could the model in use evaluate studies of a narrow specialty (e.g., neuroblastoma), whose published literature is thin and sometimes informal, as compared with data-rich fields (e.g., melanoma or breast cancer)?

### Watermarking

Could LLM-generated review content carry some embeddable marker that would make its provenance verifiable, supporting transparent reporting without requiring reviewers to self-police?

### Data-privacy certification

Should an independent third party audit and certify the privacy practices, and perform independent benchmarking, of LLM agents before they are added to a publisher’s whitelist, rather than relying on vendor claims alone, including for general-purpose tools used under institutional agreements?

### Author control

What mechanisms would let authors meaningfully exercise a right to know or object to involvement of LLM agents in reviewing their work, without simply becoming a pro forma consent checkbox?

## Conclusion

The pressures on the current peer review system are structural and unlikely to reverse on their own: submissions are growing faster than the pool of willing, qualified reviewers, manuscripts are becoming too complex for any single expert to fully vet, and editorial offices have steadily shifted burden onto unpaid reviewers. Ad hoc, undisclosed use of general-purpose LLM is already filling the gap, quietly and without oversight. If left unchecked, this unmonitored status quo has the potential to erode the long-term retention of scientific knowledge.

Professional, transparent, disclosed LLM agent use in review, whatever the underlying model, will offer a more sustainable path forward than either outright prohibition or silent, unregulated adoption. It can relieve reviewers of the most tedious, error-prone verification work while leaving judgments about novelty, significance, and overall scientific value where they belong: with human experts, and with appropriate regard for what authors themselves want from the process. That outcome will not happen by default. It requires publishers, editors, reviewers, and authors to act now, rather than waiting for a system already showing signs of strain to break down further.

## Statement of Terminology

On Sept. 29, 2026, the Federal Government Executive Order 14434, *Inaugurating the Era of Super Intelligence*, directs agencies to use “Super Intelligence” (SI) in place of “Artificial Intelligence” (AI). Since SI is an umbrella term spanning many methods, such as rule-based systems, classical machine learning, computer vision, etc, for the required technical precision in this commentary, we use **large language model (LLM) agent** to name the specific underlying technology.

## Disclosure

The authors developed the concept of this commentary and wrote all the narratives. The LLM of Claude (Opus 5.5) was used to revise the statement and search for references. All the references have been validated.

## Supplementary Material

Reviewer comments
