# Supplementary material for: LLM tool use in manuscript review: Toward guidance and transparency
Source: Life Sci Alliance. 2026 Nov 10;10(1):e202603913. doi: 10.26508/lsa.202603913 (PMC13752405; doi:10.26508/lsa.202603913)
Supplement: Supplementary file 1 [file LSA-2026-03913_review_history.pdf]

LLM Tool Use in Manuscript Review: Toward Guidance and Transparency

Chi-Ping Day, Besa Xhabija, and Vishaka Gopalan
DOI: https://doi.org/10.26508/lsa.202603913

Corresponding author(s): Chi-Ping Day, National Cancer Institute

Review Timeline:

Submission Date:	2026-09-17
Editorial Decision:	2026-10-06
Revision Received:	2026-10-14
Editorial Decision:	2026-10-22
Revision Received:	2026-10-23
Accepted:	2026-10-29

Scientific Editor: Tim Fessenden

Transaction Report:

(Note: With the exception of the correction of typographical or spelling errors that could be a source of ambiguity, letters and reports are not edited. The original formatting of letters and referee reports may not be reflected in this compilation.)

No full Peer Review Process File is available with this article, as it was decided not to make the review process public in this case.

October 6, 2026

Re: Life Science Alliance manuscript #LSA-2026-03913

Dr. Chi-Ping Day
National Cancer Institute
Cancer Data Science Laboratory
Building 15C1
NIH-Bethesda
Bethesda, Maryland 20892

Dear Dr. Day,

Thank you for submitting your commentary entitled "Learning from Asimov: Guiding the AI Tool Use in Manuscript Review" to Life Science Alliance. Your manuscript has been discussed among the editorial team, which regrettably required some time to complete. We are pleased to invite your piece for publication following revision to strengthen and focus its central points, and resolve some minor logical and formatting issues.

While you are revising your manuscript, please also attend to the below editorial points to help expedite the publication of your manuscript. Please direct any editorial questions to the journal office.

To upload the revised version of your manuscript, please log in to your account: https://lsa.msubmit.net/cgi-bin/main.plex
You will be guided to complete the submission of your revised manuscript and to fill in all necessary information. Please get in touch in case you do not know or remember your login name.

A. THESE ITEMS ARE REQUIRED FOR REVISIONS

-- A cover letter indicated your responses to the above points.

-- An editable version of the final text (.DOC or .DOCX) is needed for copyediting (no PDFs).

-- High-resolution figure, supplementary figure and video files uploaded as individual files: See our detailed guidelines for

preparing your production-ready images, https://www.life-science-alliance.org/authors

-- Summary blurb (enter in submission system): A short text summarizing in a single sentence the study (max. 200 characters including spaces). This text is used in conjunction with the titles of papers, hence should be informative and complementary to the title. It should describe the context and significance of the findings for a general readership; it should be written in the present tense and refer to the work in the third person. Author names should not be mentioned.

B. MANUSCRIPT ORGANIZATION AND FORMATTING:
Full guidelines are available on our Instructions for Authors page, https://www.life-science-alliance.org/authors
*
IMPORTANT: It is Life Science Alliance policy that if requested, original data images must be made available. Failure to provide original images upon request will result in unavoidable delays in publication. Please ensure that you have access to all original microscopy and blot data images before submitting your revxxision.*
W
e hope that our comments will be helpful towards strengthening your intriguing and timely commentary. Thank you for your contribution to Life Science Alliance. We are looking forward to receiving your revised manuscript.

Sincerely,
Tim Fessenden
Life Science Alliance

October 22, 2026

RE: Life Science Alliance Manuscript #LSA-2026-03913R

Dr. Chi-Ping Day
National Cancer Institute
Cancer Data Science Laboratory
Building 15C1
NIH-Bethesda
Bethesda, Maryland 20892

Dear Dr. Day,

Thank you for submitting your revised Commentary entitled "LLM Tool Use in Manuscript Review: Toward Guidance and Transparency". This revised version includes several changes that we suggested to make your argument more clear, and to better align with the concerns of our readership. We would be happy to publish your piece in Life Science Alliance pending the final revisions outlined below and final edits included in the attached document.

-Please add the X and Bluesky handles of your host institute/organization, as well as your own, and/or one of the authors, in our system.
-Please rename "About the authors" to the Authors' Contributions.
-Please label the conflict of interest statement as "Conflict of Interest".
-You are welcome to compose an abstract, but we leave this to your discretion.

Regarding terminology, we note that your institution has imposed regulations on use of the term "AI". While we disagree with this policy, we are happy to accommodate your suggested alternative. Please ensure your manuscript no longer refers to "AI" if indeed that is your wish, and please ensure you utilize "LLM tool"/"LLM tools" consistently.

Please upload the following materials to our submission system by logging in to your account: https://lsa.msubmit.net/cgi-bin/main.plex
You will be guided to complete the submission of your revised manuscript.

These items are required for acceptance.

-- An editable version of the final text (.DOC or .DOCX) is needed for copyediting (no PDFs).

-- Summary blurb (enter in submission system): A short text summarizing in a single sentence the study (max. 200 characters including spaces). This text is used in conjunction with the titles of papers, hence should be informative and complementary to the title. It should describe the context and significance of the findings for a general readership; it should be written in the present tense and refer to the work in the third person. Author names should not be mentioned.

The license to publish form must be signed before your manuscript can be sent to production. A link to the license to publish form will be sent to the corresponding author only. Please take a moment to check your funder requirements.

Thank you for your attention to these final processing requirements. Please revise and format the manuscript and upload materials within 7 days.

Thank you for this interesting contribution, we look forward to publishing your Review in Life Science Alliance.

Sincerely,

Tim Fessenden
Scientific Editor
Life Science Alliance

October 29, 2026

RE: Life Science Alliance Manuscript #LSA-2026-03913RR

Dr. Chi-Ping Day
National Cancer Institute
Cancer Data Science Laboratory
Building 15C1
NIH-Bethesda
Bethesda, Maryland 20892

Dear Dr. Day,

Thank you for submitting your Commissioned Commentary entitled "LLM Tool Use in Manuscript Review: Toward Guidance and Transparency". It is a pleasure to let you know that your manuscript is now accepted for publication in Life Science Alliance.

Your manuscript will now progress through copyediting and proofing. Please attend to these final edits by making changes during the proofing process.

Terminology: We do recognize that NIH policy has unfortunately prohibited your use of the more widely accepted term "AI". We invite you to consider using "LLM agent" in the text wherever you feel it is appropriate.

Title: Given the above issue with terminology we suggest changing your title to:
"LLM Agents in Manuscript Review: Toward Guidance and Transparency" or, if you wish:
"LLM Tools in Manuscript Review: Toward Guidance and Transparency"

We appreciate the inclusion of an abstract, and we suggest minor edits for clarity so that it reads as follows:
The peer review system is straining under rising submissions, growing manuscript complexity, and a shrinking reviewer pool. Meanwhile an increasing number of researchers now use large language models (LLMs) to when composing peer reviews, often without disclosure and against journal policies. This erodes trust in peer review and risks deteriorating the quality and integrity of research publications. We argue for professionalization and transparency rather than prohibition, and propose guidelines built on three aspects: model selection and data protection, human guidance and accountability, and publication integrity. These principles offer a sustainable path forward, leaving scientific judgment where it belongs; that is, with human experts.

The final published version of your manuscript will be deposited by us to PubMed Central upon publication.

IMPORTANT: If you will be unreachable at any time, please provide us with the email address of an alternate author. Failure to respond to routine queries may lead to unavoidable delays in publication.

Scheduling details will be available from our production department. You will receive proofs shortly before the publication date. Only essential corrections can be made at the proof stage so if there are any minor final changes you wish to make to the manuscript, please let the journal office know now.

If you have any questions or any issues arise in production, please feel free to contact me through the journal office:
contact@life-science-alliance.org

Thanks once again for this interesting contribution, we look forward to publishing it in Life Science Alliance.

Sincerely,

Tim Fessenden
Scientific Editor
Life Science Alliance
